# Supplementary material for: Immune and Microbial Signatures Associated with PD-1 Blockade Sensitivity in a Preclinical Model for HPV+ Oropharyngeal Cancer
Source: Cancers (Basel). 2024 May 30;16(11):2065. doi: 10.3390/cancers16112065 (PMC11171047; doi:10.3390/cancers16112065)
Supplement: Supplementary file 1 [file cancers-16-02065-s001.zip › cancers-2993990-supplementary.pdf]

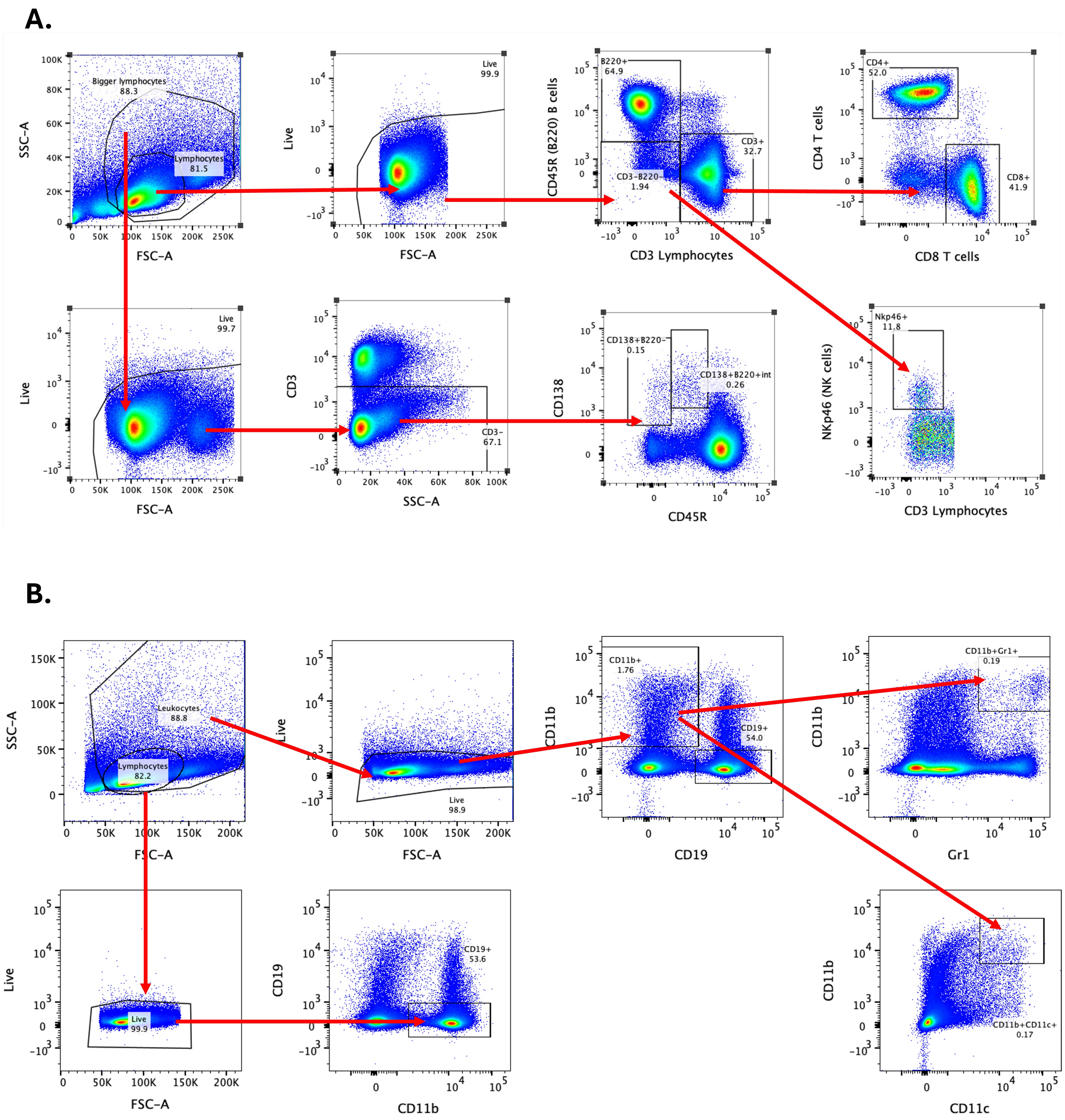

Supplementary Figure S1: Flow cytometry gating strategy used to identify the different cell subsets using the FlowJo program for the antibody panel, identifying T cells, B cells, and NK cells **(panel A)**, as well as the antibody panel identifying myeloid, dendritic, myeloid-derived suppressor cells (MDSCs) and B cells **(panel B)**.

Experimental group	Number of samples	Average of Reads	StdDev of Reads	Average of OTUs	StdDev of OTUs
1_Naive	9	12886.22222	26011.0445	231.2222222	57.95640124
2_No_Treatment	7	24823.57143	31737.52311	278.5714286	68.76010748
3_Responder	6	27886.16667	16561.49821	758.1666667	564.3053842
4_Non-responder	5	32041	3418.893608	499.6	309.412831
**Grand Total**	**27**	**22861.59259**	**23509.15738**	**410.2962963**	**351.1746021**

Supplementary Table S1. Microbial sample description in mice cohort. Summarizing the number of reads and OTUs for each mice experimental group. The metadata table includes all analyzed variables used for the QIIME2 and Microbiome Analyst platforms.

		Bacterial 16S Analyses		
			Figure 4A	Figure 4B
			KW p-value	PERMANOVA p-value
	**Experimental group 1**	**Experimental group 2**		
**HPV+ Oropharyngeal carcinoma**	Naïve	No treatment	0.560	0.536
	Naïve	Responder	0.288	0.001
	Naïve	Non Responder	0.013	0.002
	No treatment	Responder	0.317	0.003
	No treatment	Non Responder	0.291	0.016
	Responder	Non Responder	0.201	0.017

Supplementary Table S2. Pairwise statistical Kruskal-Wallis (KW) and PERMANOVA tests for alpha and beta diversity plots from main Figure 4.

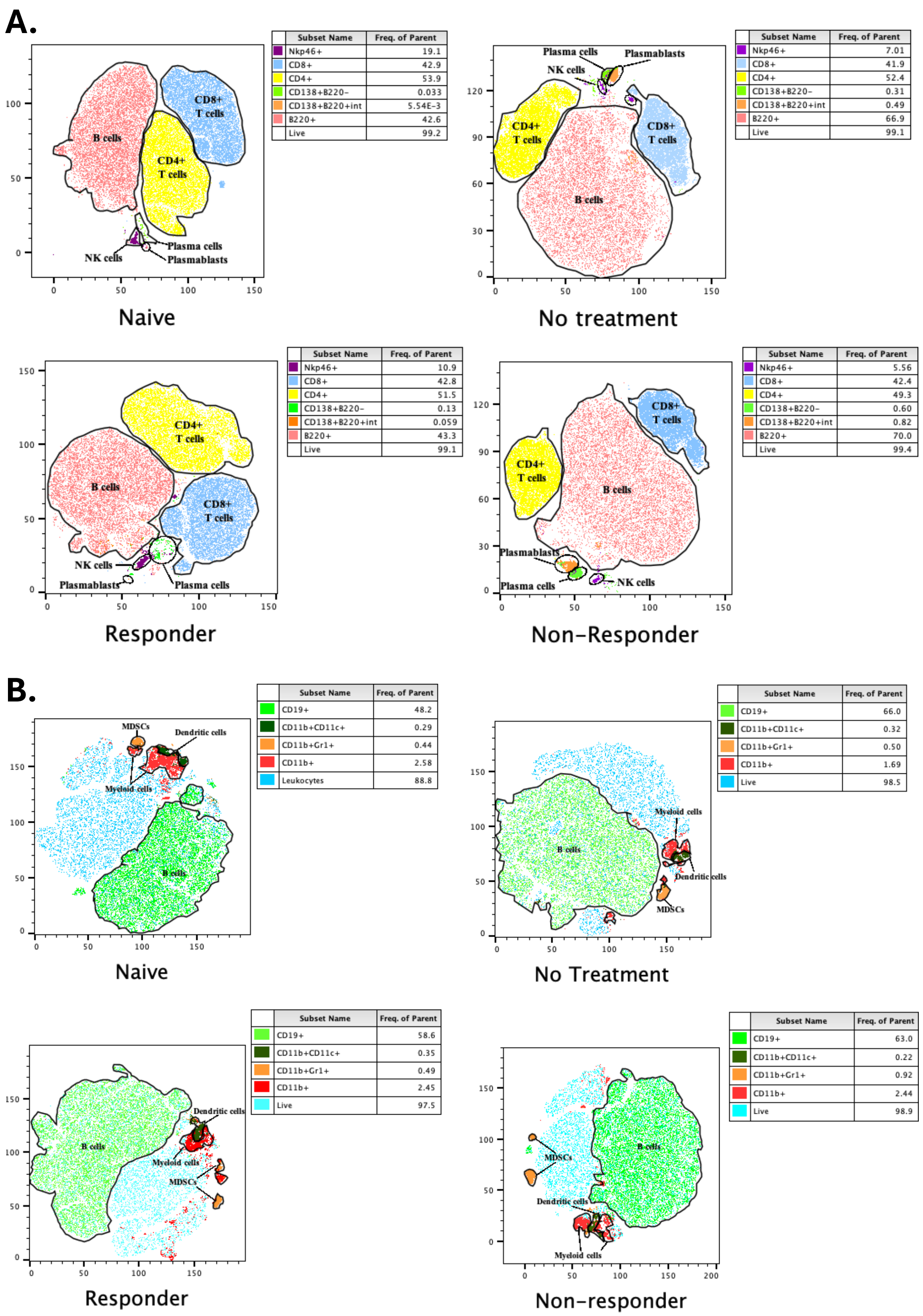

Supplementary Figure S2: Representative tSNE plots spacially depicting the distribution of immune cell subsets in tdLN tissue: CD4+ and CD8+ T cells, NK cells (NKp46), B220 + B cells, CD138+B220int plasma cells, and CD138+B220- plasmablasts **(A)**, and CD11b+ myeloid cells, CD11b+ CD11c+ dendritic cells, CD11+Gr1+ MDSCs, and CD19+ B cells **(B).**

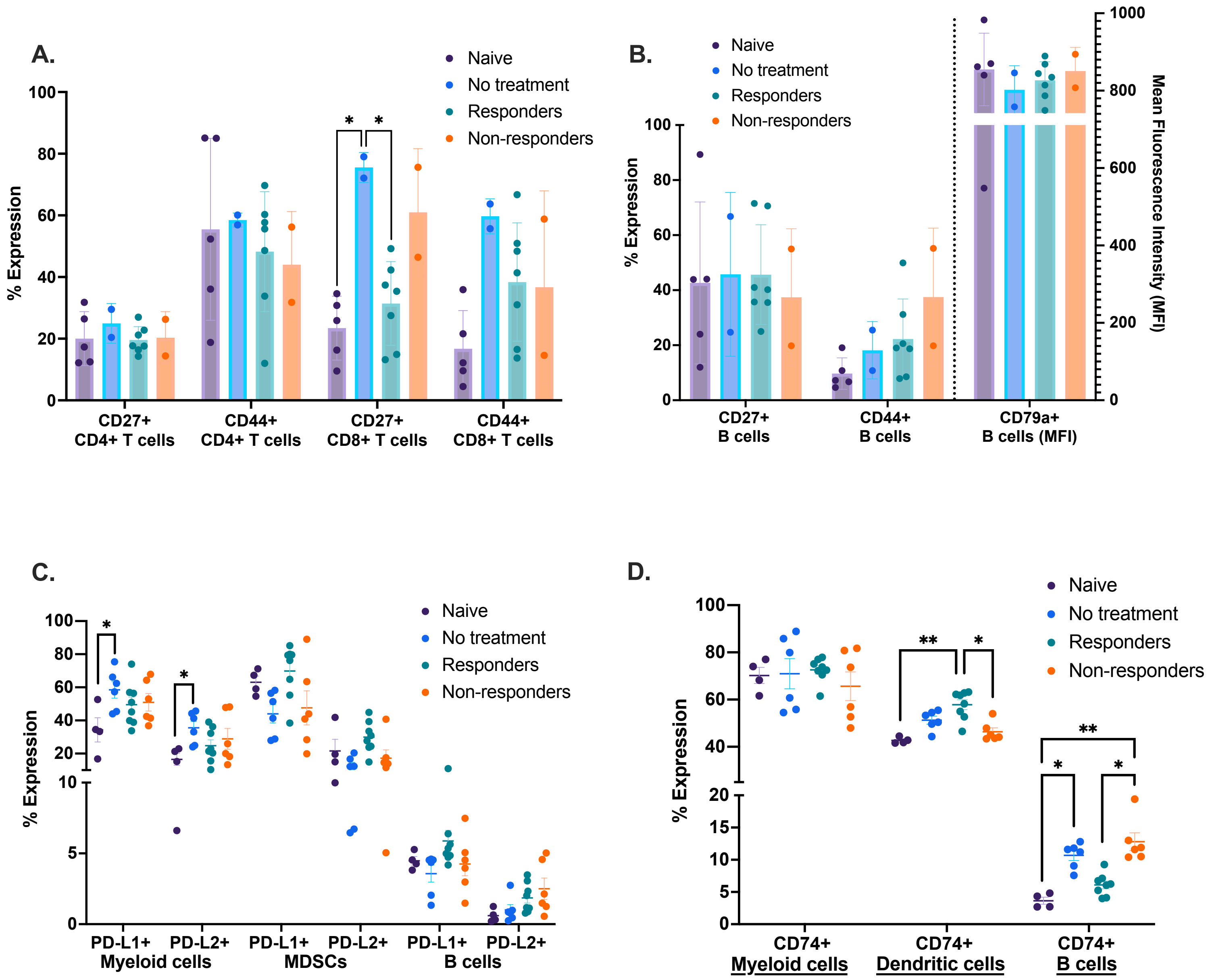

Supplementary Figure S3. Tumor expression of cell activation-associated proteins and inhibitory molecules. Tongues and tumors samples from C57BL/6 mEER implanted mice and treated with anti-PD-1 were analyzed with flow cytometry for the expression of known biomarker proteins associated with cancer therapy response and resistance in other cancers. Results depict the percent expression in tongues and tumor tissue of activation proteins CD27 and CD44 by CD4+ and CD8+ T cells **(A)**, percent expression of CD27 and CD44, and mean fluorescence intensity (MFI) of the protein CD79a by B cells **(B)**, percent expression of inhibitory molecules PD-L1 and PD-L2 by myeloid cells, myeloid-derived suppressor cells (MDSCs) and B cells **(C)**, and percent expression of CD74 by myeloid, dendritic cells, and B cells **(D)**. A Shapiro-Wilk test was performed to determine the normality of the distributions for each variable. Statistical significance of the normal distributions was determined with a two-way ANOVA, while distributions that didn't display normality were analyzed with a Kruskal-Wallis test. ****p<0.00001, ****p<0.00001, ***p<0.0001, and *p<0.01). Note: All NKp46 immune protein expression data is collected from a single 25 mice experiment.

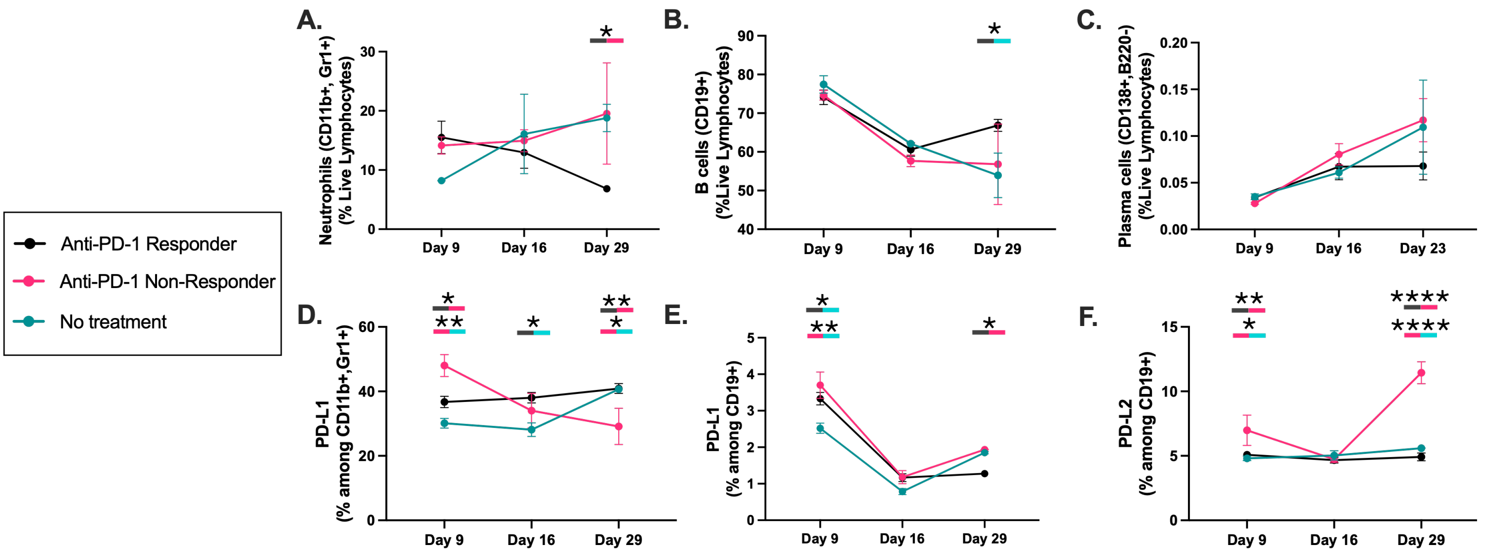

Supplementary Figure S4. Whole blood flow cytometry analysis of immune cells and immunosuppressive markers before, during, and after therapy characterizes response. Mouse whole blood was collected on days 9 (before therapy), 16 (during therapy), and 29 (after therapy) and analyzed using flow cytometry to determine the frequencies of neutrophils, B cells, and plasma cells (A-C) and percent expression of immunosuppressive molecules PD-L1 by neutrophils and B cells, and PD-L2 by B cells (D-F). Statistical analysis according to normality showed ****$p<0.00001$, ***$p<0.0001$, **$p<0.001$, and *$p<0.01$.
